# Bone Health, Body Composition and Physiological Demands in 70–85-Year-Old Lifelong Male Football Players

**DOI:** 10.3390/sports11100205

**Published:** 2023-10-18

**Authors:** Domenico Martone, Daniela Vitucci, Annamaria Mancini, Georgios Ermidis, Jeppe Panduro, Loretta Francesca Cosco, Morten Bredsgaard Randers, Malte Nejst Larsen, Magni Mohr, Pasqualina Buono, Peter Krustrup

**Affiliations:** 1 Department of Economics, Law, Cybersecurity and Sports Sciences, University Parthenope, 80035 Naples, Italy; 2 CEINGE-Biotecnologie Avanzate Franco Salvatore S.c.a.r.l, 80131 Naples, Italy; daniela.vitucci@assegnista.uniparthenope.it (D.V.); annamaria.mancini@uniparthenope.it (A.M.); germidis1990@gmail.com (G.E.); pasqualina.buono@uniparthenope.it (P.B.); 3 Department of Movement Sciences and Wellbeing, University Parthenope, 80133 Naples, Italy; lorettafrancesca.cosco001@studenti.uniparthenope.it; 4 Department of Sports Science and Clinical Biomechanics, SDU Sport and Health Sciences Cluster (SHSC), University of Southern Denmark, 5230 Odense, Denmark; jepanduro@health.sdu.dk (J.P.); mranders@health.sdu.dk (M.B.R.); mnlarsen@health.sdu.dk (M.N.L.); mmohr@health.sdu.dk (M.M.); pkrustrup@health.sdu.dk (P.K.); 5 Centre of Health Sciences, Faculty of Health, University of Faroe Islands, FO-100 Tórshavn, Faroe Islands

**Keywords:** body composition, lifelong football training, elderly, bone health, physiological demand

## Abstract

The effects of lifelong football training on bone health, body composition and physiological demands were evaluated. A total of 20 veteran football players (VPG; 73.4 ± 3.7 years) and 18 untrained age-matched men (CG; 75.6 ± 4.2 years) were enrolled. Whole-body and regional dual-energy X-ray absorptiometry scans of arms, legs, proximal femur and lower spine (L1-L4) were recorded in all participants. We observerd higher bone mineral density (BMD) in the whole-body, arms and femoral regions and higher bone mineral content (BMC) in the legs and lower spine compared to the CG (*p* < 0.05), also higher total lean body mass (*p* < 0.05) and lower total body fat percentage (*p* < 0.05), were found. No differences in food habits were evidenced between the VPG and the CG, as evaluated using 3-day food records. Resting heart rate (RHR), blood pressure (BP) and activity profile during a football match were recorded using a global positioning system only in the VPG. The mean heart rate (HR)of theoretical maximal HR (ThHRmax), and peak of ThHRmax were 83.9 ± 8.6% and 98.6 ± 10.2%, respectively; the mean of total distance covered was 3666 ± 721 m, and the means of accelerations and decelerations were 419 ± 61 and 428 ± 65, respectively. Lifelong participation in football training improves regional BMD and BMC in legs, femur and lumbar spine compared to the CG. A high number of intense actions in term of HR and accelerations and decelerations suggests an elevated energy expenditure that in turn correlates to the healthier body composition observed in the VPG compared to the CG.

## 1. Introduction

The global life expectancy is considerably increased, with a growing number of elderlies in the general population. By 2030, one in six people in the world will be aged 60 years or over. The number of people aged 80 years or older is expected to triple between 2020 and 2050 to reach 426 million [1]. Aging is a physiological process characterized by a progressive decline in biological functions and a deterioration process in cells and organs, eventually leading to death [2,3,4,5].

Beyond biological changes, aging is often associated with other life conditions such as retirement, daily living involving a more sedentary lifestyle due to a large amount of sitting time during the day, a nutritional regimen that is not adequate and increased leisure time [1]. All these biological and environmental conditions can lead to loss of muscle mass, poor bone health, metabolic and cardiovascular diseases and in some cases chronic inflammation, cognitive decline, neurodegenerative disorders and deteriorated social and mental wellbeing [6,7,8,9]. In particular, the prevalence of osteoporosis and osteoporotic fractures is expected to rise dramatically in the future in the aged population [9,10]. Moreover, it has been estimated that approximately 20% of all patients with a hip fracture do not survive longer than one year from diagnosis and more than 50% never completely regain their previous functional status leading to loss of independence, need for long-term care and reduced quality of life [10,11].

Physical activity and exercise training have been shown to have positive effects, delaying decline in most of the above-mentioned age-related factors [12,13,14,15,16]. One form of exercise that has been extensively studied, due to its worldwide ubiquity and popularity, is football, and the health benefits of participating in recreational football are extensively demonstrated [16].

Football is a multi-component sport combining high-intensity training (HIIT), endurance and strength training elements with more than 100 high-intensity runs and hundreds of specific intense actions such as dribbles, shots, tackles, turns and jumps in a 60 min training session. Moreover, recreational football training elicits average heart rates of 80–85% of maximal heart rate (HRmax) during a 60 min session, with 15–50% of total training time spent in the highest aerobic training zone, also above 90% HRmax in older people [17,18,19,20,21,22]. Such stimuli induce substantial improvements in muscular (endurance and muscular strength) and systemic (higher VO_2_max and healthier metabolic profile), reducing the risk of metabolic and cardiovascular diseases (CVD) compared to untrained subjects also among the elderly [21].

In addition, football training with several turns, jumps and sprints, and with accelerations and decelerations causes high impact forces on the bones. It has been demonstrated that regular football training performed for at least 12–64 weeks improves the osteogenesis in healthy and unhealthy subjects ranging from 9 to 73 years [19,20]. A recent metanalysis has also reported that regular football training results in a reduction in total body fat mass and an increase in total lean body mass [19,20].

Despite more than 250 million active football players being present worldwide [22], only a few studies have described the effects of lifelong football training on physical, physiological and metabolic characteristic’s profile and the effects on longevity in veteran football players (VPG) compared to age-matched untrained subjects (CG) [23,24,25,26].

In such studies, it has been observed that during matches the VPG showed a mean heart rate (HR) of around 80% of HRmax, with high energy expenditure and had healthier body composition [23,24,25,26,27,28,29,30]. Furthermore, it has been observed that the VPG had a better whole-body and leg BMD (body mineral density) and cardiovascular function than the CG [23,24].

No studies have evaluated the potential osteogenic effect of lifelong football training in other different body regions such as lumbar spine and femur. Moreover, until now the physiological demands and body composition have not been fully characterized in the VPG.

Thus, the first aim of the present controlled cross-sectional study was to analyze the effects of lifelong football training on body composition, BMD and body mineral content (BMC) in the whole-body and in different regions including arms, legs and lower spine in the VPG compared to the CG. The secondary aim was to analyze the physiological demands (e.g., heart rate, number of accelerations and decelerations, distance covered and maximal speed) of a football match in the VPG.

## 2. Materials and Methods

### 2.1. Participants

Thirty-eight men were recruited for this study, including 20 for VPG and 18 for CG, through The Danish Football Association (Dansk Boldspil-Union; DBU), along with coaches in local football clubs near Odense, Denmark. CG was recruited through advertisements in social media and local newspapers near Odense (Denmark). VPG men were aged 73.4 ± 3.7 years (range: 70–82 years) and had played football regularly (≥1 session per week) for more than 40 years (64.5 ± 4.1), had for the last 10 years participated in more than 26 football matches per year and were still active prior to testing, whereas CG men were aged 75.6 ± 4.2 years (70–84 years) and had not participated in regular physical activity and sport during their life since participating in activities during their school time. All participants were non-smoking with no chronic diseases; however, mild hypertension or hyperlipidemia were not exclusion criteria. Three and six members of VPG and CG, respectively, took blood-pressure-lowering medication, while one and four participants in these groups, respectively, took cholesterol-lowering medications. Furthermore, four and three subjects (VPG and CG, respectively) were medicated with blood thinners. Exclusion criteria were unbalanced diets such as vegan, vegetarian, ketogenic, ergogenic and vitamins supplementation (except vitamin D), antibiotics and anti-inflammatory drugs. All subjects were fully informed about the experimental procedures and possible discomforts or risks associated with participation before giving their written informed consent. The study was conducted in accordance with the guidelines of the Declaration of Helsinki and was approved by regional ethics committee for Southern Denmark (ID: S-20180165; approved on 6 March 2019).

### 2.2. Study Design

**Figure 1:**
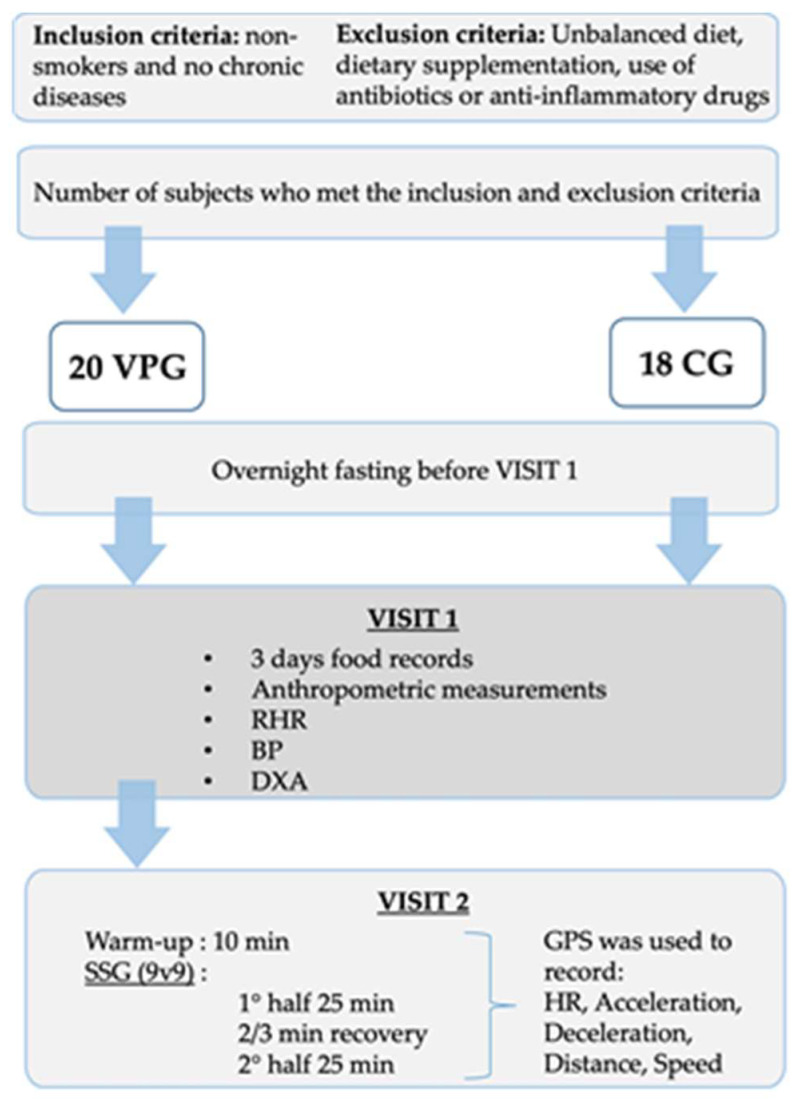
Flowchart of study design. Abbreviations: SSG, small-sided games; GPS, global positioning system; HR, heart rate; RHR, resting heart rate; BP, blood pressure; DXA, dual-energy X-ray absorptiometry.

The participants reported to the laboratory on two separate occasions. On visit 1, anthropometric measurements, resting heart rate (RHR), blood pressure (BP) and dual-energy X-ray absorptiometry (DXA) were carried out for both VPG and CG. All measurements were performed in the morning between 7 and 9 am after an overnight fast by trained experienced personnel. Moreover, to estimate the daily food intake, participants filled out a questionnaire (3-day food records). Records were processed using Winfood software version 3.14.3 (Medimatica S.u.r.l., Colonnella, Italy). On visit 2, a football match organized in two 25 min halves separated by 2–3 min of recovery (standing still or slow walking) was completed. The football match, performed at 11 am and preceded by a 10-min warm-up period, was organized as a small-sided game (9v9) on a 45 m wide and 65 m long natural grass pitch with a total of 50 min playing time and all participants serving as goalkeeper, rotating every ~3 min. HR and physical activity profile were measured using HR monitors and global positioning system (GPSPolar Team Pro, Polar Electro Oy, Kempele, Finland) during the match. Two subjects did not take part in the football match for personal reasons. Figure 1 shows a flowchart of study design.

### 2.3. Anthropometric Measurements

Body weight and height were measured to the nearest 0.1 kg and 0.1 cm, respectively, with subjects in bare feet and light clothing using standardized equipment (InBody 230 analyzer, Seoul, Republic of Korea) and body mass index (BMI) was calculated as weight in kilograms divided by the square of height in meters (kg/m^2^).

### 2.4. BP and RHR

All measurements were performed after 48 h without training for VPG, in a quiet room under standardized conditions and after an overnight fast. Before measuring, the subjects rested for at least 15 min in supine position, and BP was measured six times using an automated BP monitor (M7, OMRON, Vernon Hills, IL, USA) on the left upper arm. The average of six measurements was used for the analysis. RHR was determined as the lowest average value obtained using the monitor over a 1-min period.

### 2.5. Body Composition, BMD and BMC Assessment

All participants underwent a dual femur and whole-DXA scan in accordance with standard procedures (Prodigy Advance, Lunar Corporation, Madison, WI, USA). To evaluate regional BMD and BMC in both legs, whole-body and lower spine (L1, L2, L3 and L4) the scan was segmented in accordance with standard procedures. Total lean body mass (kg), total body fat mass (kg), total body fat percentage (%), leg lean mass (kg), android body fat (%) and gynoid body fat (%) were also assessed using DXA scanning. All analyses were performed using enCORE software version 15 (Lunar Corporation, Madison, WI, USA). The effective radiation dose for dual femur and whole-body scan in total was 10.84 mSv.

### 2.6. Heart Rate and Physical Demands during a Football Training Session

Physical demands and HR data were collected using Polar Team Pro units (Polar Electro Oy, Kempele, Finland) with GPS sampling at a 10 Hz frequency, 200 Hz tri-axial accelerometer, gyroscope, magnetometer and HR monitor, and analyzed using proprietary software. The 10 Hz GPS measurements with subsequent data fusion with inertial measurement units using Kalman filter have been shown to provide valid and reliable data [31,32]. HR and physical demands data were analyzed during the whole match excluding the half-time break. The HR data were expressed as mean HR while peak HR was determined as the highest value reached for each subject during the match. In addition, Tanaka et al. [33] equation (208 − 0.7 × age) was used to predict the theoretical maximum HR (ThHRmax) in each participant. The percentage time spent in different HR zones was categorized into five different zones as follows: <120 bpm, 120–140 bpm, 140–160 bpm, 160–180 bpm and >180 bpm. Physical demands variables included for the analysis were: total distance (m), relative distance (m/min), maximal speed (km/h), average speed (km/h), number of sprints > 18 km/h (n) and distance covered in different speed zones: 0–8.99 km/h, 9–12.99 km/h, 13–15.99 km/h, 16–19.99 and >20 km/h. Total number of accelerations and decelerations (n) and total number of accelerations and decelerations by zones: 0.5–0.99 m/s^2^, 1–1.99 m/s^2^, 2–2.99 m/s^2^ and >3 m/s^2^ were also recorded.

### 2.7. Statistical Analyses

Before experimental testing, G*power 3.1 software [34] was used to determine the number of participants required per group to have a statistical power of 0.80 or higher with an effect size of 0.5 at the significance level alpha = 0.05. According to Skoradal et al. [35], a minimum fold change of 2% in BMD and BMC, due to osteogenic effect of football training, would be biologically and clinically relevant, thus, we calculated that a sample size of 15 VPG and 15 CG was needed for our study.

Comparisons between groups were determined with the Student’s *t*-test for all normally distributed variables or with Mann–Whitney test for non-parametric variables. In the match, the differences in physiological and physical demands variables in VPG were determined using a one-way repeated measures analysis of variance (ANOVA) with Bonferroni post hoc testing to identify the points of difference. In case of not normalized variables, Friedmann test (with Kendall’s concordance coefficient W) followed by pairwise comparison (Durbin–Conover) were performed. Effect size (ES) was also calculated and interpreted as suggested by Hopkins et al. [36]: <0.2 trivial; >0.2 and<0.6 small; >0.6 and <1.2 moderate; >1.2 and <2.0 large and >2.0 very large. All data are expressed as mean ± standard deviation (SD) and as medians (Mdn; IQR) for those non-normally distributed. The level of significance was set at *p* < 0.05 and all analyses were performed using Jamovi software (version 2.2.5.0).

## 3. Results

### 3.1. Whole-Body and Regional BMD and BMC

Whole-body and regional BMD and BMC assessments are reported in Table 1 and Table 2, respectively.

Whole-body BMD and BMC were 13.2% higher in the VPG compared to the CG (*p* < 0.001, ES = 1.660 and *p* = 0.002 ES = 1.085), respectively. The BMD was 7.7% higher (*p* = 0.03 and ES = 0.734) in the VPG while the BMC was similar in the arms (*p* = 0.145 and ES = 0.484) compared to the CG, respectively. Furthermore, the VPG showed 16.8% and 17.9% higher leg BMD and BMC compared to the CG (*p* < 0.001 and ES = 1.520 and *p* < 0.001 and ES = 1.192), respectively. Higher L1, L2, L3 and L4 vertebrae BMD (13.6%,15.0%, 19.4% and 21.6%, *p* = 0.051 and ES = 0.986; *p* = 0.009 and ES = 0.898; *p* = 0.003 and ES = 1.040 and *p* < 0.001 and ES = 1.290), respectively; and 21.3%, 21.0%, 28.4% and 33.2% higher L1,L2,L3 and L4 vertebrae BMC (*p* = 0.006 and ES = 0.964; *p* = 0.014 and ES = 0.843; *p* = 0.003 and ES = 1.056 and *p* < 0.001 and ES = 1.468), respectively, were also observed in the VPG compared to the CG. In both legs, the VPG showed a 8.1–22.6% higher BMD in total proximal femur and all femoral regions (*p* < 0.05 and ES = 0.585–1.245) compared to the CG (Table 1).

### 3.2. Anthropometrics, Body composition, Daily Caloric and Nutrient Intake, BP and RHR

There were no statistically significant differences in age, height, total body mass and BMI between the VPG and the CG. Compared to the CG, the VPG had 3.7 kg higher total lean body mass (*p* = 0.043 and ES = 0.673), 1.6 kg higher leg lean mass (*p* = 0.019 and ES = 0.447) and 3.5 kg lower total body fat mass (*p* = 0.047 and ES = 0.057), together with 4.7% lower total body fat percentage (*p* = 0.005 and ES = 0.977), 6.7% lower android body fat percentage (*p* = 0.009 and ES = 0.896) and 5.5% lower gynoid body fat percentage (*p* = 0.001 and ES = 1.169). There were no differences in BP and RHR observed between the VPG and the CG (Table 3). The 3-day food records questionnaire was completed by 19 members of the VPG and 9 of the CG, respectively. There were not significant differences (*p* > 0.05) between groups in daily caloric and nutrients intake (Table 4).

**Figure 2:**
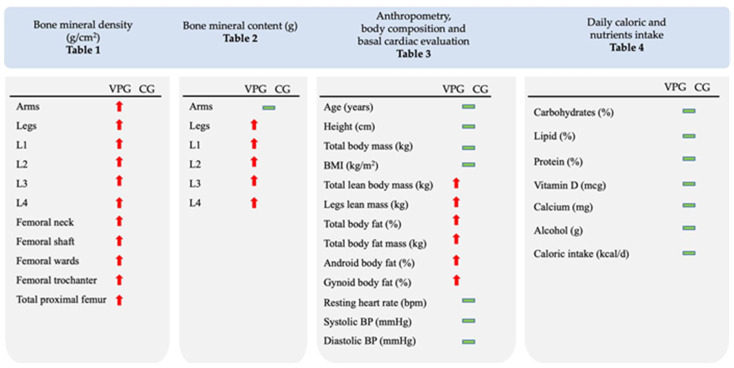
Summary of differences between VPG and CG for various parameters analyzed. The red arrows indicate a statistically significant increase (*p* < 0.05) in VPG compared to CG and the green horizontal bars indicate no difference between groups. Numerical and statistical values are given in the respective tables.

A summary of differences between the VPG and the CG for the various parameters analyzed is reported in Figure 2.

### 3.3. Physiological Demands during a Football Match

Mean HR and peak HR measured during the football match for the VPG were 131 ± 15 bpm and 155 ± 18 bpm, respectively. The participant’s mean HR of ThHRmax, and peak of ThHRmax were 83.9 ± 8.6% (with range of between 72.0 and 98.7% of their ThHRmax) and 98.6 ± 10.2% (with a range between 83.7 and 120.7%), respectively. The maximal and average speeds were 18.6 ± 3.0 km/h and 3.9 ± 0.7 km/h, respectively (Table 5).

**Figure 3:**
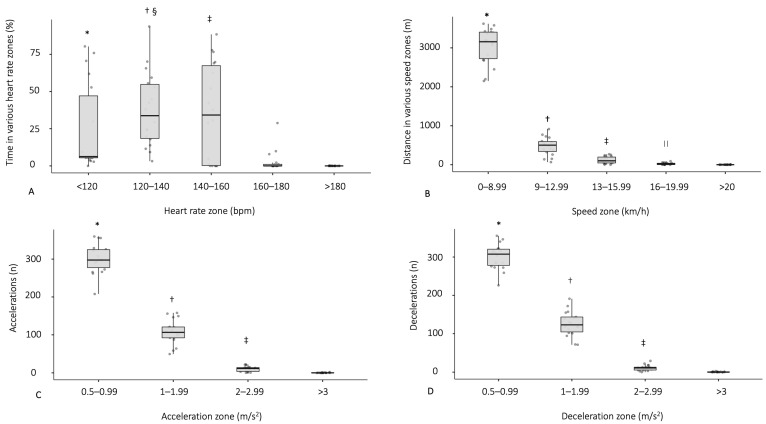
Percentage of effective match time spent in various heart rate zones (**A**), distance covered in different speed zones (**B**), number of accelerations (**C**) and decelerations (**D**) in different acceleration and deceleration zones measured during veteran football players’ (VPG; n = 18) participation in the study. The inside line represents the median; the box, the interquartile range; the solid gray circles, the individual value and the whiskers, the highest and lowest values. (**A**): * < 120 bpm vs. 160–180 bpm, * < 120 bpm vs. >180 bpm (*p* < 0.001); ^†^ 120–140 bpm vs. 160–180 bpm, ^†^ 120–140 bpm vs. >180 bpm (*p* < 0.001); ^‡^ 140–160 vs. 160–180 bpm, ^‡^ 140–160 bpm vs. >180 bpm (*p* < 0.001); ^§^ 120–140 bpm vs. <120 bpm (*p* = 0.03); (**B**): * 0–8.99 km/h vs. 9–12.99 km/h; * 0–8.99 km/h vs. 13–15.99 km/h, * 0–8.99 km/h vs. 16–19.99 km/h and * 0–8.99 km/h vs. >20 km/h (*p* < 0.001); ^†^ 9–12.99 km/h vs. 13–15.99 km/h, ^†^ 9–12.99 km/h vs. 16–19.99 and ^†^ 9–12.99 km/h vs. >20 km/h (*p* < 0.001); ^‡^ 13–15.99 km/h vs. 16–19.99, ^‡^ 13–15.99 km/h vs. >20 km/h (*p* < 0.001); ^||^ 16–19.99 vs. >20 km/h (*p* < 0.001); (**C**,**D**): * 0.5–0.99 m/s^2^ vs. 1–1.99 m/s^2^, * 0.5–0.99 m/s^2^ vs. 2–2.99 m/s^2^ and * 0.5–0.99 m/s^2^ vs. >3 m/s^2^ (*p* < 0.001); ^†^ 1–1.99 m/s^2^ vs. 2–2.99 m/s^2^ and ^†^ 1–1.99 m/s^2^ vs. >3 m/s^2^ (*p* < 0.001); ^‡^ 2–2.99 m/s^2^ vs. >3 m/s^2^ (*p* < 0.001).

Significant differences in the time percentage spent in different HR zones during the 50 min of game time (χ^2^ (4) = 40.2, *p* < 0.001 and W = 0.56) were evidenced. Post hoc analysis revealed that most of the game time percentage was spent at an intensity between 120 and 140 bpm (Mdn = 33.5 and IQR [18.2–54.2]) and 140–160 bpm (Mdn = 33.5 and IQR [0.0–67.2]) (Figure 3A). The total distance covered during the match was 3666.2 ± 721.5 m (Table 5). Significant differences in distances covered in different speed zones (χ^2^ (4) = 70.2, *p* < 0.001 and W = 0.97) were also evidenced. Post hoc analysis revealed that most of the distance covered during the match was performed between 0 and 8.99 km/h (Mdn = 3157.5 and IQR [2724.0–3404.0.2]) (Figure 3B).

Significant differences in the total numbers of accelerations and decelerations in different zones (χ^2^ (3) = 53.7, *p* < 0.001 and W = 0.99 and χ^2^ (3) = 53.7, *p* < 0.001 and W = 0.99, respectively) were evidenced in the VPG during the match. Post hoc analysis revealed that most of the accelerations and decelerations, at least 300, were performed at 0.5–0.99 m/s^2^ (Mdn = 297.5 and IQR [277.5–325.0] and Mdn = 307.5 and IQR [278.5–320.5], and at least 100 were performed between 1 and 1.99 m/s^2^, respectively (Figure 3C,D).

## 4. Discussion

The present study aimed to evaluate the effects of lifelong (at least 40 years) football training on BMC and BMD in whole-body and in different body regions, including legs, the femur region, the lower spine and the arms in the 70–85-yr-old VPG compared to the CG, and as a secondary aim, it analyzed the physiological demands of a football match in the VPG. We found that the VPG with regular participation in football training had significantly higher BMD and BMC values index compared to the CG with a broad effect ranging from 6.3% to 33.2% in whole-body, arms, legs, proximal femur and lower spine. The VPG also had a healthier body composition, with higher total lean body mass and a lower percentage of total, android and gynoid body fat compared to the CG.

Our results are in line with Hagman et al. [26], who evaluated the BMD and BMC at regional, whole-body, leg and proximal femur values in a VPG. The authors evidenced that a lifelong VPG aged 65–80 years had 9.4% to 10.2% and 6.4% to 7.2% higher BMD and BMC in whole-body and legs, respectively, and ~7% to 13% higher BMD in total proximal femur and all femoral regions except the trochanter compared to age-matched untrained subjects.

Until now, the mechanisms linking lifelong football training to BMD and BMC improvement have not been completely understood. It has been suggested that the bone adaptions and BMC increases may be due to changes in bone turnover markers (BTMs) following repeated bouts of exercise [16]. An increased BMD value associated with circulating osteogenic markers (e.g., osteocalcin, P1NP and CTX-1) has been reported in many cross-sectional and intervention studies after acute, short and long term regular training involving subjects at different ages and with different health conditions [37,38,39]. Moreover, it also has been demonstrated that type, intensity and total amount of aerobic exercise are involved in the BTMs in response to the exercise [20,40,41]. In the present study, more than 35% of the total match time was spent at an average of >90% of ThHRmax with a mean of 419 and 428 accelerations and decelerations, respectively. BTMs across a longer time of training (>40 year and lifelong) could explain the osteogenic effect induced by football observed in the VPG compared to the CG.

In cross-sectional studies it is difficult to understand if regular football training may be the sole cause of the osteogenic adaptions or whether they are due to many confounding factors, such as nutritional habits. For instance, vitamin D and calcium intake are very important in bone formation/remodeling mainly in older people [42,43]. Since both groups showed no significant differences in nutritional habits as well as in vitamin D and calcium intake, higher values of BMD and BMC observed in the VPG compared to the CG suggest that football training had an effective role in maintaining lifelong bone health.

Moreover, our results are in line with those obtained in many intervention studies evidencing that in untrained elderly men, BMD increased by up to 1.8% and 5.4% in the proximal femur after 4 and 12 months of regular football training, respectively [37,38,44]. Mohr et al. [38] evidenced that higher femoral shaft and trochanter BMD values, ranging from 1.7% to 2.4%, respectively, were observed in middle-aged women after 15 weeks of football training. Also, Helge et al. [37] reported an increase in BMD in the left and right tibia ranging from 2.6% to 2.1%, respectively, after 14 weeks of football training in premenopausal middle-aged untrained women. Further, studies in rats have shown that load-induced increases in BMD and BMC of 5–12% improve the bone strength by 64–87%, reducing the risks of bone fractures and premature death due to post operative complications and comorbidities [16]. Since it was estimated that the decline in BMD is about 0.5% per year for men older than 40 years [44], the values observed in the VPG in the present study suggest that participation in regular football training helps to keep lifelong bone health, preventing the age-related impairment.

Other cross-sectional and interventional studies involving lifelong football and handball players, healthy elderly males and senior endurance athletes, evidenced healthier body composition with a reduction in total e regional fat mass in lifelong football players compared to untrained controls [23,26]. Hagman et al. [26] found a lower total body fat percentage (∼4%) in lifelong football players than in age-matched controls with no differences observed in total lean body mass. Schmidt et al. [23] reported a ∼6%, 9% and 6% lower, total, android and gynoid body fat percentage, respectively, in lifelong football players compared to elderly untrained controls.

The improvement in body composition in the VPG, evidenced in this study, may be due, in part, to the intense activity observed during the football match with more than half of the time session (70%) performed within a HR range 76–89% of individual ThHRmax. At this intensity, the exercise has been shown to be equally effective as longer periods of moderate continuous intensity training in terms of reduction in total fat mass and abdominal adipose tissue associated with improved insulin sensitivity and glycemic control [45,46,47].

Few studies have described the physiological and physical demands of football training in the elderly [23,24,25,26], while no study has been evaluated in a VPG that has played football regularly for more than 40 years. Our results showed that in the VPG, the mean HR of ThHR was about 84% with 108 accelerations and 126 decelerations detected ranging from 1 to 1.99 m/s^2^ and about 18 min of total time were spent jogging and low-speed running in agreement with previous studies [24,25,48,49]. Time spent at the intensities of exercise observed in our study and in the above-mentioned studies is markedly higher than the minimum suggested for cardiovascular fitness by the American College of Sports Medicine (55–65% HRmax) and is associated with improvements in systolic blood pressure, glucose tolerance and healthier body composition [50,51,52].

Considering high intensity in terms of mean HR during the match, football can be considered a global game that requires high energy expenditure. In fact, a mean energy expenditure of ~1300–1600 kcal, and a total match day energy expenditure of ~3500 kcal in elite football players, were evidenced [53]. Moreover, it has been estimated that one session of 60 min per week of recreational football requires an energy expenditure of ~600 kcal, which represents almost 50% of energy expenditure suggested by international guidelines for traditional physical activity [54]. Football training also positively affects the expression of markers associated with health and cardiorespiratory fitness. In particular, recently, the increased expression of muscular molecular markers involved in oxidative metabolism (i.e., AMPKα1/α2, NAMPT, TFAM and PGC1α, MyHC β isoform expression and citrate synthase) in lifelong football-trained men compared to active untrained controls has been evidenced [29]. These findings were associated with a reduction in total body fat mass, an increase in total lean body mass and higher VO_2_max in trained compared to untrained elderly people [21]. Thus, lifelong football training represents a good strategy to promote not only a healthier body composition but also the general efficiency of body systems.

A limitation of this study is the inter-subjects’ variability in genetics, heritable and environmental factors that could have affected the differences in bone mass, body composition and other physiological parameters evaluated in the VPG and the CG. The other limits were the analysis of only one match to determine the activity profile, the small number of subjects examined and the absence of women. Thus, it will be interesting to expand the number of subjects, also including women, and analyze the effect of lifelong training in different types of sports on bone health and body composition.

## 5. Conclusions

The results of the present study evidenced for the first time that lifelong football training (>40 years) with a high number of intense actions interspersed with low-intensity actions improves regional BMD and BMC mainly in the lumbar spine and femur and induces a healthier body composition in the VPG compared to the CG. We also evidenced that a high number of intense actions in term of HR and accelerations and decelerations suggests an elevated energy expenditure that in turn correlates to the healthier body composition observed in the VPG compared to the CG.

## Figures and Tables

**Table 1:** Bone mineral density (g/cm^2^) in whole-body, arms, legs, lower spine and proximal femur in veteran football players (VPG) and age-matched untrained subjects (CG) participating in the study (mean ± SD).

	VPG (n = 20)	CG (n = 18)	Delta (%)	*p*-Value	ES	95% CI	
						Lower	Upper
Whole-body	1.344 ± 0.131	1.187 ± 0.072	13.2	<0.001	1.660	0.820	2.470
Arms	1.047 ± 0.096	0.972 ± 0.108	7.7	0.030	0.734	0.048	1.400
Legs	1.423 ± 0.165	1.218 ± 0.089	16.8	<0.001	1.520	0.708	2.300
L1	1.196 ± 0.156	1.053 ± 0.131	13.6	0.005	0.986	0.257	1.690
L2	1.365 ± 0.299	1.187 ± 0.155	15.0	0.009	0.898	0.191	1.590
L3	1.460 ± 0.265	1.223 ± 0.184	19.4	0.003	1.040	0.311	1.740
L4	1.523 ± 0.241	1.252 ± 0.219	21.6	<0.001	1.290	0.520	2.030
Femoral neck							
Right	0.916 ± 0.135	0.847 ± 0.092	8.1	0.048	0.585	0.094	1.256
Left	0.950 ± 0.154	0.827 ± 0.102	14.9	0.010	0.919	0.190	1.634
Femoral shaft							
Right	1.167 ± 0.162	1.048 ± 0.159	11.4	0.030	0.745	0.047	1.420
Left	1.192 ± 0.155	1.057 ± 0.162	12.8	0.016	0.854	0.133	1.554
Femoral wards							
Right	0.755 ± 0.137	0.616 ± 0.071	22.6	<0.001	1.245	0.476	1.993
Left	0.760 ± 0.148	0.631 ± 0.080	20.4	<0.001	1.055	0.306	1.780
Femoral trocanter							
Right	0.935 ± 0.142	0.810 ± 0.111	15.4	0.006	0.966	0.240	1.675
Left	0.937 ± 0.120	0.814 ± 0.103	15.1	0.003	1.090	0.336	1.824
Total proximal femur							
Right	1.030 ± 0.130	0.916 ± 0.115	12.4	0.009	0.917	0.198	1.620
Left	1.047 ± 0.129	0.918 ± 0.115	14.1	0.004	1.039	0.293	1.764

Abbreviations: L1 to L4, lumbar spine vertebrae; ES, effect size and CI, confidence interval.

**Table 2:** Bone mineral content (g) in whole-body, arms, legs and lower spine in veteran football players (VPG) and age-matched untrained subjects (CG) participating in the study (mean ± SD).

	VPG (n = 20)	CG (n = 18)	Delta (%)	*p*-Value	ES	95% CI	
						Lower	Upper
Whole-body	3248.0 ± 411.0	2869.2 ± 264.4	13.2	0.002	1.085	148.67	608.80
Arms	446.1 ± 63.9	419.6 ± 41.78	6.3	0.145	0.484	9.530	62.41
Legs	1297.4 ± 196.7	1100.1 ± 121.4	17.9	<0.001	1.192	88.21	306.37
L1	19.4 ± 3.71	16.0 ± 3.22	21.3	0.006	0.964	1.030	5.71
L2	22.5 ± 4.94	18.6 ± 4.11	21.0	0.014	0.843	0.84	6.86
L3	26.7 ± 6.54	20.8 ± 4.34	28.4	0.003	1.056	2.22	9.61
L4	30.5 ± 5.72	22.9 ± 4.63	33.2	<0.001	1.468	4.23	11.12

Abbreviations: L1 to L4, lumbar spine vertebrae; ES, effect size and CI, confidence interval.

**Table 3:** Anthropometry, body composition and basal cardiac evaluation in veteran football players (VPG) and age-matched untrained subjects (CG), enrolled in the study (mean ± SD).

Variable (Units)	VPG (n = 20)	CG (n = 18)	Delta(%)	*p*-Value	ES	95% CI	
						Lower	Upper
Age (years)	73.4 ± 3.7	75.6 ± 4.2	2.9	0.060	0.554	0.113	1.214
Height (cm)	174.1 ± 4.8	176.4 ± 5.2	1.3	0.930	0.464	0.197	1.114
Total body mass (kg)	81.9 ± 8.3	81.6 ± 11.3	0.4	0.160	0.027	−0.610	0.668
BMI (kg/m^2^)	27.0 ± 2.7	26.2 ±3.6	3.0	0.450	0.248	−0.409	0.882
Total lean body mass (kg)	57.3 ± 4.4	53.6 ± 7.1	6.9	0.043	0.673	0.002	1.347
Leg lean mass (kg)	19.2 ± 1.8	17.6 ± 2.7	9.1	0.019	0.447	−0.003	1.339
Total body fat (%)	25.4 ± 5.7	30.1 ± 3.8	4.7	0.005	0.977	0.250	1.662
Total body fat mass (kg)	21.0 ± 6.6	24.5 ± 5.4	14.3	0.047	0.057	−1.228	0.095
Android body fat (%)	33.3 ± 7.8	40.0 ± 7.1	6.7	0.009	0.896	−0.189	1.583
Gynoid body fat (%)	23.8 ± 5.4	29.3 ± 3.8	5.5	0.001	1.169	−0.411	1.880
Resting heart rate (bpm)	60.7 ± 10.1	62.6 ± 11.9	3.0	0.595	0.174	−0.811	0.467
Systolic BP (mmHg)	137.8 ± 14.5	135.3 ± 15.1	1.8	0.598	0.173	−0.468	0.810
Diastolic BP (mmHg)	80.3 ± 8.4	78.4 ± 9.0	2.4	0.508	0.217	−0.426	0.855

**Table 4:** Daily caloric and nutrients intake of veteran football players (VPG) and age-matched untrained subjects (CG) participating in the study (mean ± SD).

Variable (Units)	VPG (n = 19)	CG (n = 9)	*p*-Value
Carbohydrates (%)	40.0 ± 8.6	39.8 ± 3.9	0.947
Lipid (%)	39.5 ± 7.9	40.0 ± 4.9	0.859
Protein (%)	17.2 ± 3.7	18.6 ± 3.8	0.362
Vitamin D (mcg)	2.8 ± 3.2	2.4 ± 1.8	0.885
Calcium (mg)	526.1 ± 276.8	603.5 ± 170.0	0.449
Alcohol (g)	19.6 ± 17.2	23.3 ± 20.0	0.977
Caloric intake (kcal/d)	1878.7 ± 491.1	1823.8 ± 493.7	0.785

**Table 5:** Physiological and physical demands measured during a match involving veteran football players (VPG, n = 18) enrolled in the study.

Variable (Units)	Mean ± SD (Min–Max)
Mean HR (bpm)	131 ± 15 (108–157)
Peak HR (bpm)	155 ± 18 (126–191)
ThHR max (bpm)	157 ± 2 (155–159)
Mean HR of ThHR max (%)	83.9 ± 8.6 (72.0–98.7)
Peak of ThHRmax (%)	98.6 ± 10.2 (83.7–120.7)
Total distance (m)	3666 ± 721 (2260–4539)
Relative distance (m/min)	61 ± 12 (38–76)
Maximal speed (km/h)	18.6 ± 3.0 (12.8–26.9)
Average speed (km/h)	3.9 ± 0.7 (2.6–4.8)
Number of sprints > 18 km/h (n)	0.4 ± 0.6 (0.0–2.0)
Total number of accelerations (n)	419 ± 61 (294–529)
Total number of accelerations by zone (n):	
0.5–0.99 m/s^2^	300 ± 38 (208–360)
1–1.99 m/s^2^	108 ± 32 (49–158)
2–2.99 m/s^2^	10 ± 7 (0–22)
>3 m/s^2^	0 ± 1 (0–2)
Total number of decelerations (n)	428 ± 65 (286–552)
Total number of decelerations by zone (n):	
0.5–0.99 m/s^2^	300 ± 33 (226–355)
1–1.99 m/s^2^	126 ± 32 (71–191)
2–2.99 m/s^2^	11 ± 7 (0–28)
>3 m/s^2^	1 ± 1 (0–3)

Abbreviations: HR, heart rate and ThHR, theorical heart rate.

## Data Availability

Data generated or analyzed during this study are available from the corresponding author upon reasonable request.
